# Evaluation of genetic risk scores for lipid levels using genome-wide markers in the Framingham Heart Study

**DOI:** 10.1186/1753-6561-3-s7-s46

**Published:** 2009-12-15

**Authors:** Stephen R Piccolo, Ryan P Abo, Kristina Allen-Brady, Nicola J Camp, Stacey Knight, Jeffrey L Anderson, Benjamin D Horne

**Affiliations:** 1 Department of Biomedical Informatics, School of Medicine, University of Utah, 26 South 2000 East, Salt Lake City, Utah 84112, USA; 2 Cardiovascular Department, Intermountain Medical Center, 5121 South Cottonwood Street, Salt Lake City, Utah 84107, USA; 3 Cardiology Division, School of Medicine, University of Utah, 30 North 1900 East, Salt Lake City, Utah 84132, USA

## Abstract

**Background:**

Multiple single-nucleotide polymorphisms have been associated with low-density lipoprotein cholesterol (LDL-C), high-density lipoprotein cholesterol (HDL-C), and triglyceride (TG) levels. In this paper, we evaluate a weighted and an unweighted approach for estimating the combined effect of multiple markers (using genotypes and haplotypes) on lipid levels for a given individual.

**Methods:**

Using data from the Framingham Heart Study SHARe genome-wide association study, we tested genome-wide genotypes and haplotypes for association with lipid levels and constructed genetic risk scores (GRS) based on multiple markers that were weighted according to their estimated effects on LDL-C, HDL-C, and TG. These scores (GRS-LDL, GRS-HDL, and GRS-TG) were then evaluated for associations with LDL-C, HDL-C, and TG, and compared with results of an unweighted method based on risk-allele counts. For comparability of metrics, GRS variables were divided into quartiles.

**Results:**

GRS-LDL quartiles were associated with LDL-C levels (*p *= 2.1 × 10^-24^), GRS-HDL quartiles with HDL-C (*p *= 5.9 × 10^-22^), and GRS-TG quartiles with TG (*p *= 5.4 × 10^-25^). In comparison, these *p*-values were considerably lower than those for the associations of the unweighted GRS quartiles for LDL-C (*p *= 3.6 × 10^-7^), HDL-C (*p *= 6.4 × 10^-16^), and TG (*p *= 4.1 × 10^-10^).

**Conclusion:**

GRS variables were highly predictive of LDL-C, HDL-C, and TG measurements, especially when weighted based on each marker's individual association with those intermediate risk phenotypes. The allele-count GRS approach that does not weight the GRS by individual marker associations was considerably less predictive of lipid and lipoprotein measures when the same genetic markers were utilized, suggesting that substantially more risk-associated genetic marker information is encapsulated by the weighted GRS variables.

## Background

Low-density lipoprotein cholesterol (LDL-C), high-density lipoprotein cholesterol (HDL-C), and triglycerides (TG) are accepted risk predictors for coronary artery disease (CAD) [1,2]. Recently published genome-wide association studies reported novel loci for LDL-C, HDL-C, and TG, while also confirming several previously described loci [3-5]. Those studies replicate each other for many of the loci; however, it is unclear how the combined effect of these markers for a given patient should be estimated most effectively.

The genetic risk score (GRS) model is an approach for evaluating multiple related markers simultaneously in association testing for clinical phenotypes such as lipid levels. A recent publication reported an unweighted, integer-based GRS calculated as the sum of risk alleles associated with LDL-C and HDL-C [6]. We previously introduced the concept of aggregating polygenic information in a GRS weighted by the estimated effect of each marker on intermediate risk phenotypes [7]. For this investigation, we hypothesized that the weighted GRS approach would be more powerful than an integer-based GRS because it accounts for variability in the effect of each marker on the phenotype and thus may better represent the complex physiology that drives changes in lipid and lipoprotein levels.

Risk scores proposed thus far, including our own, are based on modelling the contributions of multiple, individual single-nucleotide polymorphisms (SNPs). We hypothesized that haplotypes also are relevant for identifying association evidence and that such biologically relevant markers are overlooked in single-marker analyses. Hence, we also performed haplotype-based association testing in regions where individual SNPs were below the threshold for genome-wide significance and included these markers in GRS models.

Using markers that individually were associated with lipid/lipoprotein levels, we constructed weighted and unweighted GRS metrics for individuals in the Framingham Heart Study and estimated the ability of each composite measure to predict lipid levels.

## Methods

### GAW16 data from Framingham Heart Study

For this analysis, SNP data from the Genetic Analysis Workshop 16 (GAW16) Problem 2 (real phenotype data) were utilized. These data arose from the Framingham Heart Study SHARe genome-wide association study that evaluated DNA markers for a large subset of the Framingham Heart Study participants using the Affymetrix GeneChip^® ^Human Mapping 500 k Array Set. The data were hosted by and acquired through the NIH dbGAP tool. This investigation was in compliance with the Data Use Agreement required for access to this data set as well as with the requirements of the University of Utah's Institutional Review Board.

### Clinical data elements

The Framingham data set contained phenotypes for 373 individuals from the original cohort, 2,760 from the offspring cohort, and 3,997 from the third generation. Data elements for each participant were selected from the first available study exam for which total cholesterol, HDL-C, and TG levels were available. Because LDL-C measurements were not provided, values were estimated for each participant using the equation:

Of the 7,130 participants, 397 were excluded because of missing measurements for cholesterol, HDL-C, or TG at all available study visits.

### Association testing

A genome-wide analysis was undertaken to test association of genotypes and haplotypes with LDL-C, HDL-C, and TG. Before this, an additional 322 individuals were excluded because they reported medication use for the treatment of dyslipidemia. Quality control also screened out SNPs and participants with <98% genotyping success and SNPs not in Hardy-Weinberg equilibrium (*p *< 0.001). After filtering these individuals, 6,411 remained for association testing.

Genotypes were tested for association with each phenotype using two randomly assigned (on a per-individual basis) subsets of the study population--one with *n *= 3,847 participants (60% of total population) and another with *n *= 2,564 participants (40% of total population). The purpose of dividing the data was to select markers that could reach significance (though not necessarily at a genome-wide level) in two samples. We performed a quantitative association test using the PLINK software [8] with default configuration settings and a significance level of *p *< 0.001. SNPs identified in this step were carried forward for use in the GRS models.

Haplotypes were constructed and tested for association with each phenotype. The PLINK software [8] was used on the full data set to identify SNPs that obtained a *p*-value between 10^-2 ^and 10^-4^. We chose this threshold range with the goal of identifying SNPs that did not reach genome-wide significance alone but that would be substantially more significant as part of a haplotype. The regions containing these SNPs were ranked using a scoring metric that increased according to the significance level of nearby SNPs (<10^-2 ^= +1, <10^-3 ^= +2, or <10^-4^= +3). After identification of an initial SNP with a *p*-value between 10^-2 ^and 10^-4^, the score for that region was increased until 15 nearby SNPs failed to reach at least a significance level of 10^-2^. Haplotype analyses were then pursued in the top ten scoring regions (which spanned ~25 kilobases and contained ~7 SNP markers). The haplotype analyses were performed using hapConstructor [9], which uses a forward-backward search algorithm to construct haplotypes and test for association. Its haplotype-mining technique starts with single SNPs and builds up haplotypes SNP-by-SNP, searching for the most significant haplotype across multiple models (dominant, recessive, and additive). The SNP sets considered need not be contiguous. It then uses a difference-of-means test statistic to test for haplotype associations and assesses significance via a Monte Carlo approach (500,000 null simulations maximum). The two most significant haplotypes for each phenotype were used in the GRS models. The full data set was used for identifying haplotypes due to the lack of a straightforward approach for combining results from two data subsets.

### Weighted GRS

GRS variables for each phenotype were calculated separately for each participant (in the full data set), including those on anti-dyslipidemia medications. TG levels were natural-log transformed. The estimated effect size for each genotype selected from the association analysis described above was calculated as the mean (median for TG) difference in lipid levels between those with the wild-type variant and those homozygote for the rare variant (effect = 0 for heterozygotes). For each marker, wild-type, heterozygote, and homozygote recessive genotype carriers received a score of -1, 0, or -1, respectively, multiplied by the marker's estimated effect size. These scores were summed into a GRS for each participant.

Due to the approximately continuous distribution of the weighted GRS values, these variables were divided into quartiles of similar sample size for the purposes of comparing association statistics with those computed for the unweighted GRS variable and to present the data in a clinically relevant framework in which thresholds are used for decision-making. Linear mixed models [10] were used to test for association of GRS variables with LDL-C, HDL-C, and TG, with family membership included in each model as a random-effects variable to adjust for pedigree membership. Multivariate analysis of variance was also used to simultaneously model the association of GRS-LDL, GRS-HDL, and GRS-TG with the dependent variables LDL-C, HDL-C, and TG to compute coefficients of determination that were comparable to the unweighted GRS.

### Unweighted GRS

The unweighted, risk-allele based GRS method [6] is calculated for each participant as the sum of risk allele counts across each marker that predicts LDL-C, HDL-C, or TG. This unweighted GRS was composed using the same markers as the weighted analysis and was evaluated on the same data set of participants. Any marker associated with multiple lipid/lipoprotein phenotypes was included only once in the summation. A value of 0, 1, or 2 was assigned to each SNP based on carriage of the same number of copies of the risk allele. These values were then summed for each participant and divided into quartiles for comparative purposes. Association of this GRS with LDL-C, HDL-C, and TG was performed as above using a linear mixed model and coefficients of determination calculated using multivariate analysis of variance.

## Results

### Marker selection

For LDL-C, six SNPs reached *p *< 0.001 in both data subsets (Table 1). SNPs associated with HDL-C at *p *< 0.001 in both subsets included 11 SNPs on chromosome 8p21 that defined two haplotypes in the region 3' to the *LPL *gene. A single SNP in each of these haplotypes was sufficient to explain >90% of the haplotypic variance, and thus represented the haplotype in the GRS. Nine SNPs had *p *< 0.001 in both subsets for TG. Of these SNPs, rs599839 had the strongest individual association with any phenotype (LDL-C), obtaining a *p*-value of 9.5 × 10^-13 ^in the first data subset and 2.2 × 10^-16 ^in the entire data set.

**Table 1:** SNPs associated with LDL-C, HDL-C, and TG in GWAS, with the gene that they are in or near

			*p*-Value			
				
Gene	SNP	Chr	Subset 1	Subset 2	Overall	Effect size (mg/dL)^a^
*LDL-C*						
PSRC1^b^	rs599839	1p13	9.5 × 10^-13^	1.6 × 10^-6^	2.2 × 10^-16^	-16.7
CELSR2^b^	rs4970834	1p13	5.6 × 10^-7^	6.8 × 10^-5^	1.1 × 10^-9^	-10.3
PSMA5	rs17586966	1p13	1.8 × 10^-5^	3.6 × 10^-4^	2.8 × 10^-7^	-8.8
MTPN	rs1365360	7q33	4.2 × 10^-4^	7.8 × 10^-4^	1.4 × 10^-6^	7.7
DCDC1/5	rs158584	11p14	2.7 × 10^-4^	5.3 × 10^-4^	2.3 × 10^-7^	7.1
RORA	rs782918	15q22	3.2 × 10^-4^	6.3 × 10^-4^	3.1 × 10^-7^	-9.8
*HDL-C*						
LPL^c^	rs17482753	8p21	5.4 × 10^-10^	9.2 × 10^-6^	5.5 × 10^-13^	11.1
LPL	rs10503669	8p21	1.1 × 10^-9^	4.9 × 10^-6^	2.5 × 10^-13^	12.4
LPL	rs17410962	8p21	7.4 × 10^-8^	2.9 × 10^-5^	6.9 × 10^-11^	8.5
LPL	rs17489268	8p21	1.1 × 10^-9^	3.5 × 10^-6^	6.6 × 10^-15^	4.5
LPL	rs17411024	8p21	4.6 × 10^-8^	1.5 × 10^-5^	1.6 × 10^-11^	5.5
LPL	rs17411031	8p21	2.1 × 10^-9^	3.2 × 10^-6^	1.3 × 10^-14^	4.5
LPL	rs17411126	8p21	3.3 × 10^-9^	7.7 × 10^-6^	6.1 × 10^-14^	4.4
LPL	rs765547	8p21	4.3 × 10^-9^	6.1 × 10^-6^	4.6 × 10^-14^	4.5
LPL	rs11986942	8p21	1.1 × 10^-6^	4.0 × 10^-4^	3.1 × 10^-9^	3.6
LPL	rs1837842	8p21	4.9 × 10^-9^	1.8 × 10^-6^	1.7 × 10^-14^	4.6
LPL	rs1919484	8p21	5.9 × 10^-9^	2.7 × 10^-6^	3.2 × 10^-14^	4.6
CETP^c^	rs9989419	16q13	1.1 × 10^-4^	4.6 × 10^-4^	8.2 × 10^-7^	-3.6
LIPG^b^	rs7240405	18q21	1.0 × 10^-4^	6.9 × 10^-4^	5.3 × 10^-7^	-3.6
LIPG	rs4939883	18q21	2.9 × 10^-5^	6.2 × 10^-4^	1.7 × 10^-7^	-3.6
ACAA2^b^	rs6507945	18q21	4.6 × 10^-4^	1.0 × 10^-4^	6.8 × 10^-7^	-3
*Triglycerides*						
SUMF1	rs317608	3p26	5.7 × 10^-4^	1.4 × 10^-4^	4.0 × 10^-7^	26
RASGEF1B	rs4382026	4q21	6.3 × 10^-4^	3.6 × 10^-5^	5.3 × 10^-9^	22
BAZ1B	rs2074755	7q11	1.4 × 10^-5^	2.0 × 10^-4^	3.9 × 10^-10^	-13
LPL^c^	rs17489268	8p21	1.5 × 10^-6^	1.6 × 10^-4^	9.8 × 10^-12^	-13
LPL	rs17411031	8p21	9.3 × 10^-7^	1.6 × 10^-4^	6.0 × 10^-12^	-13
LPL	rs17411126	8p21	1.4 × 10^-6^	2.0 × 10^-4^	1.3 × 10^-11^	-13
LPL	rs765547	8p21	3.6 × 10^-6^	4.9 × 10^-4^	6.9 × 10^-11^	-13
LPL	rs1837842	8p21	4.8 × 10^-6^	3.4 × 10^-4^	7.1 × 10^-11^	-13
LPL	rs1919484	8p21	1.7 × 10^-5^	3.3 × 10^-4^	3.6 × 10^-10^	-12

^a^Effect sizes given here are the difference in the mean (TG: median) between the homozygote recessive genotype and homozygote wild-type

^b^See Kathiresan et al. [5]

^c^See Purcell et al. [8]

For all three phenotypes, a number of regions existed where haplotype associations were an order of magnitude more significant than any single SNP in the region. For HDL-C, seven haplotypes achieved *p *< 10^-4^, and one haplotype attained a *p*-value of 8.0 × 10^-6^. For LDL-C, two haplotypes reached *p *< 10^-4^. For TG, two haplotypes reached *p *≤ 6.0 × 10^-4^. For the latter two phenotypes, no haplotype attained a *p*-value less than 10^-5^. The results for the top two haplotypes associated with each phenotype are listed in Table 2.

**Table 2:** The two most significant haplotypes^a ^for each phenotype

Phenotype	Chr	SNPs	Haplotype	Model	*p*-Value	Effect size^b^
HDL-C	5	rs922556, rs10070979	1-2	Dominant	8.0 × 10^-6^	-16.1 mg/dL
HDL-C	3	rs2724721, rs384828	2-2	Dominant	5.1 × 10^-4^	-1.9 mg/dL
LDL-C	6	rs316024, rs576075, rs614564, rs533452	2-1-1-1	Dominant	5.3 × 10^-5^	3.4 mg/dL
LDL-C	7	rs4947934, rs4947936	2-1	Additive	6.0 × 10^-5^	16.4 mg/dL
TG	3	rs7645357, rs4685635	2-2	Additive	5.0 × 10^-4^	-9.0 mg/dL
TG	10	rs603269, rs475945	2-2	Additive	6.0 × 10^-4^	3.0 mg/dL

^a^SNPs with *p *< 10^-2 ^and *p *> 10^-4 ^were used to construct haplotypes and associations were tested in hapConstructor [9]

^b^Mean (TG: median) difference for listed haplotype vs. most common haplotype

### GRS association

Table 3 shows the results of testing for association between the weighted GRS and lipid/lipoprotein levels. The GRS for each phenotype was associated far more strongly than the association that was observed for the phenotype with any individual marker (compare with Table 1). Some weak cross-association was seen for GRS-LDL with HDL-C and TG, for GRS-HDL with TG, and for GRS-TG with HDL-C.

**Table 3:** Weighted GRS results and GRS associations with lipid/lipoprotein levels^a^

					Mean (LDL-C & HDL-C)/median (TG) in GRS by quartile				
						
GRS	Min	Max	Mean	S.D.	1	2	3	4	*p*-Value
GRS-LDL	-34.8	15	-2.69	6.8					
LDL-C					107.2	115.8	118.1	122.2	2.1 × 10^-24^
HDL-C					53.9	53.5	52.3	53.0	0.07
TG					81.0	83.0	83.0	81.0	0.04
GRS-HDL	-10.2	17	-1.28	3.5					
LDL-C					116.1	116.3	117.2	115.1	0.6
HDL-C					50.5	51.7	54.3	56.2	5.9 × 10^-22^
TG					83.0	87.0	84.0	76.0	3.4 × 10^-6^
GRS-TG	-26.0	31	-2.10	8					
LDL-C					115.1	116.6	117.4	115.5	0.43
HDL-C					54.9	54.1	51.5	52.5	7.7 × 10^-9^
TG					72.0	79.0	86.0	94.0	5.4 × 10^-25^

^a^Results of association testing using a linear mixed model with family membership included in the model as a random-effects variable. Phenotype values are in mg/dL

Table 4 shows the association results for the unweighted GRS. It is of note that the association with LDL-C would not have achieved genome-wide significance if it had been a single marker. The lower predictive ability of the unweighted GRS is understandable given the integer-based composition and the lower range of 6-20. (See Table 4. Compare this with the range of values for GRS-LDL, GRS-HDL, and GRS-TG in Table 3).

**Table 4:** Unweighted GRS (sum of risk alleles carried, as defined in literature reports [6]) and its association with lipid/lipoprotein levels^a^

	Number of risk alleles carried				Mean (LDL-C & HDL-C)/median (TG) in GRS by quartile				
GRS	Min	Max	Mean	S.D.	1	2	3	4	*p*-Value
	6	20	12.8	2.1					
LDL-C					112.8	113.4	117.9	119.9	3.6 × 10^-7^
HDL-C					56.2	53.8	51.8	51.2	6.4 × 10^-16^
TG					75.0	84.0	84.0	87.0	4.1 × 10^-10^

^a^Results of association testing using a linear mixed model with family membership included in the model as a random-effects variable. Phenotype values are in mg/dL

Coefficients of determination for the unweighted GRS were *r*^2 ^= 0.007, 0.016, and 0.010 for LDL-C, HDL-C, and TG, respectively. In comparison, modelling of GRS-LDL, GRS-HDL, and GRS-TG showed *r*^2 ^= 0.025, 0.023, and 0.024 for LDL-C, HDL-C, and TG. Although low (but not much lower than a recent report using 30 SNPs [11] that showed *r*^2 ^= 0.06-0.09), *r*^2 ^was greater for the weighted GRS approach.

GRS associations using the weighted GRS methods and including the two best haplotype markers for LDL-C, HDL-C, and TG provided marked improvement (Table 5) in the association of GRS-LDL with LDL-C (*p *= 6.0 × 10^-28^) and GRS-HDL with HDL-C (*p *= 6.2 × 10^-25^), but not for GRS-TG with TG (*p *= 6.7 × 10^-23^, which is lower than the GRS that did not use haplotype markers [see Table 3]). Changes in associations due to inclusion of haplotype markers were on the order of 10^-4 ^for GRS-LDL with LDL-C, 10^-3 ^for GRS-HDL with HDL-C, and 10^1 ^for GRS-TG with TG.

**Table 5:** Weighted GRS composed from both SNP and haplotype markers^a^

					Mean (LDL-C & HDL-C)/median (TG) in GRS by quartiles				
						
GRS	Min	Max	Mean	S.D.	1	2	3	4	*p*-Value
GRS-LDL	-34.8	21	-1.59	7.3					
LDL-C					107.7	115.2	119.1	122.7	6.0 × 10^-28^
HDL-C					54.3	53.5	53.2	53.2	0.22
TG					79.0	81.0	83.0	79.0	0.055
GRS-HDL	-27.0	17	-1.74	3.6					
LDL-C					115.7	116.4	117.2	115.5	0.53
HDL-C					50.5	51.7	54.9	56.9	6.2 × 10^-25^
TG					84.0	84.5	83.0	75.0	5.6 × 10^-8^
GRS-TG	-32.0	31	-2.59	8.4					
LDL-C					114.5	118.2	116.8	116.2	0.07
HDL-C					55.0	53.5	52.5	52.6	4.5 × 10^-5^
TG					72.0	81.0	85.0	90.0	6.7 × 10^-23^

^a^Results of association testing using a linear mixed model with family membership included in the model as a random-effects variable. Phenotype values are in mg/dL

## Discussion

Many SNPs influence lipid and lipoprotein levels [12]. This study of GRS models illustrates the potential to stratify genetic risk for complex phenotypes by accounting for polygenic effects. Aggregating data from many markers into a single GRS variable allows genetic and biological information related to a phenotype to be condensed into a statistical metric of low dimensionality.

We identified multiple SNPs that were associated (*p *< 0.001) with lipid levels in two data subsets. Most of these attained genome-wide statistical significance in the full data set, even after a conservative Bonferroni correction. The weighted GRS variables were associated with the phenotypes at a substantially better significance level (lower *p*-value), supporting the concept that a score based on multiple SNPs may be used effectively to represent the joint contributions of components in the underlying biological pathway.

In this study, the associations with LDL-C, HDL-C, and TG were greater for the weighted GRS than for the unweighted GRS, a single composite metric based only on risk-allele carriage. GRS metrics that use only the carriage of risk alleles may account only for the presence and not the relative value of those alleles [6,13]. While shown here and previously [6,13] to have some value, in an unweighted GRS the relative contribution of individual genetic markers is ignored, thus treating each as equal in effect and potentially mis-specifying the actual risk relationship. Based on these results, our weighted method appears to provide an additional ability to extract clinically meaningful genetic information for a risk pathway and encapsulate it into a useful, low-dimension variable.

In this study, each weighted GRS showed strong association with its target phenotype. Though they likely would not supplant the need for standard serum measurements of lipid and lipoprotein levels, these GRSs potentially could be useful in identifying individuals early in life who are at increased lifetime risk, which can lead to advanced phenotypes such as CAD [1,2]. Clinicians potentially could also use the GRS information to better target medical therapies and diagnostic screening for preventive purposes. This analysis also suggests that a GRS approach may be more useful and effective at characterizing risk of coronary heart disease endpoints than individual genetic markers, although this remains to be tested.

This study has several limitations. The GRS models were evaluated on the same data set on which association testing and construction of the models were performed. This likely led to over-fitting of the models, potentially biasing significance levels for the GRS association testing. However, both the weighted and unweighted GRS models would be affected by this bias and thus the comparisons between them should remain valid.

Further, the genome-wide association analyses using PLINK were done using a naïve approach that did not account for the family structure in the Framingham Heart Study data. In a study based on these data, some of the authors report in another GAW16 paper [14] that even though a naïve approach is anti-conservative, the results from an empirical analysis would have been highly correlated with the results of this naïve approach.

## Conclusion

GRS variables aggregating genetic and intermediate risk phenotype information were highly predictive of LDL-C, HDL-C, and TG measurements, and these associations were improved with inclusion of haplotype information at loci for which SNPs had only weak associations. Summation of risk alleles in an integer-based unweighted GRS were substantially less predictive of the lipid/lipoprotein phenotypes.

## List of abbreviations used

CAD: Coronary artery disease; GAW16: Genetic Workshop Analysis Workshop 16; GRS: Genetic risk scores; HDL-C: High-density lipoprotein cholesterol; LDL-C: Low-density lipoprotein cholesterol; SNP: Single-nucleotide polymorphisms; TG: Triglyceride.

## Competing interests

The authors declare that they have no competing interests.

## Authors' contributions

SRP and RPA participated in processing data, conceiving the study design, and writing the manuscript. KA-B participated in processing data, conceiving the study design, and critically revising the manuscript. NJC participated in conceiving the study design and critically revising the manuscript. SK participated in performing statistical analysis and critically revising the manuscript. JLA participated in critically revising the manuscript. BDH participated in conceiving the study design, performing statistical analysis, and writing the manuscript. All authors read and approved the final manuscript.
